# Human Papillomavirus (HPV) and the quadrivalent HPV Vaccine among Brazilian adolescents and parents: Factors associated with and divergences in knowledge and acceptance

**DOI:** 10.1371/journal.pone.0241674

**Published:** 2020-11-12

**Authors:** Jéssica Menezes Gomes, Beatriz Machado Silva, Edige Felipe de Sousa Santos, Patricia Jane Kelly, Annielson de Souza Costa, Albertina Duarte Takiuti, Luiz Carlos de Abreu, José Maria Soares Júnior, Edmund Chada Baracat, Isabel Cristina Esposito Sorpreso

**Affiliations:** 1 Disciplina de Ginecologia, Faculdade de Medicina, Universidade de Sao Paulo, Sao Paulo, SP, Brazil; 2 Study Design and Scientific Writing Laboratory at ABC Medical School, Santo André, SP, Brazil; 3 School of Nursing and Health Studies, University of Missouri—Kansas City, Kansas City, MO, United States of America; Rudjer Boskovic Institute, CROATIA

## Abstract

**Background:**

Low national immunization coverage (44.64%) requires strengthening the vaccination campaign to improve knowledge about HPV and its vaccine among adolescents and parents/guardians. Our aim is to evaluate factors related to knowledge about HPV, its vaccine, acceptability and divergences among Brazilian adolescents and parents/guardians.

**Methods:**

A cross-sectional study was performed at a health unit of Sao Paulo University, Brazil, from 2015 to 2016. The convenience sample comprised 1047 individuals, including 74% (n = 776) adolescents and 26% (n = 271) parents/guardians, who answered a survey (knowledge about HPV, its vaccine, barriers and acceptability).

**Results:**

The main source of information for adolescents was school (39%, n = 298); for parents/guardians, it was health professionals (55%, n = 153). Parents/guardians were 2.48 times more likely than adolescents to know that HPV caused changes in the Pap smear test [RR 2.48, 95% CI 2.03–3.01 (p < 0.001)], 1.43 times likely to be aware that HPV was a sexually transmitted infection [RR 1.43, 95% CI 1.22–1.68 (p < 0.001)], and 2.77 times likely to be informed that the HPV vaccine decreased the chance of having genital warts [RR 2.77, 95% CI 2.22–2.47 (p < 0.001)]. Girls knew more about the topic than boys (RR 1.67; 95% CI 1.10–2.60); education increased parents’ knowledge [(RR 3.38; 95% CI 1.71–6.69)].

**Conclusion:**

Female adolescents and parents/guardians with a higher level of education are factors related to suitable knowledge about HPV and its vaccine among Brazilian respondents. There were differences between parents/guardians and adolescents in HPV awareness, clinical implications, vaccine knowledge and vaccine acceptance.

## Background

The persistence of human papillomavirus (HPV) infection is the leading cause of more than 90% of cervical cancer cases and is responsible for a significant fraction of other anogenital [anal (90%), vulvar (70%), vaginal (70%) and penile cancer (60%)] and oropharyngeal cancer (60%) [1]. Cervical cancer is the third most common type of cancer in women aged 15 to 44 years in Brazil with an estimated 16,298 new cases diagnosed annually in the country [2].

The quadrivalent HPV vaccine is a primary prevention strategy for cervical cancer. The vaccine is available in immunization programs worldwide in 64 countries including Brazil [3]. Free access in Brazil has been provided by the Immunization Program in the Unify Health System (UHS) since 2014 [4], initially for adolescent girls and people (men and women) living with HIV (Human Immunodeficiency Virus) and in 2017 male adolescents were included [5].

To guarantee efficient results and reduce the instance of cervical precancerous lesions vaccination coverage with all doses should exceed 80% [6] of the target audience, which, according to the Brazilian immunization program, corresponds to female adolescents from 9 to 14 years of age, male adolescents from 11 to 14 years of age, and men and women living with HIV from 9 to 26 years of age [5]. However, data show that HPV vaccine uptake for complete vaccination (two doses) in female adolescents was 44.6%, in Brazil, and 60.8% in the state of Sao Paulo in 2015 [7], and both figures are declining.

There are few records in Latin American literature on adherence to the HPV vaccine after its implementation. What has been published highlights the lack of knowledge about HPV infection among Latin Americans, which is related to low education status and economic income [8–10]. Doubts about the vaccine’s safety and efficacy, and cultural myths, such as HPV vaccination stimulating the onset of sexual life, are additional barriers for vaccine acceptance [11,12]. In addition, uptake rates for other vaccines available to adolescents in the Immunization Program, such as the hepatitis B vaccine (with 85% uptake) [7], are higher than those of the HPV vaccine, which reinforces the need to explore barriers to HPV vaccine acceptance [13].

The decision-making process concerning adolescents`vaccination is influenced by parents/guardians’ lack of willingness to vaccinate their dependent [11,12,14], especially those with early vaccination age, although teenagers (over 16 years of age) in Brazil have the right to be vaccinated without parental consent [15].

The purpose of this study is to evaluate factors related to knowledge about HPV, its vaccine, acceptability and divergences among Brazilian adolescents and parents/guardians.

## Method

### Study design/Setting/Period

A cross-sectional analytic study was conducted in a basic health unit linked to the Obstetrics and Gynecology Department of the Medical School of the -University of Sao Paulo, Sao Paulo State, city of São Paulo, Brazil, from 2015 to 2016 during quadrivalent HPV vaccination implementation.

### Sample and study size

The convenience sample was composed of 1047 participants—Adolescents attending a public clinic and their accompanying parents/guardians made up the study sample. If teenagers arrived accompanied by more than one guardian, the guardians who volunteered to answer the questionnaire first was selected. The inclusion criteria were male and female adolescents, according to the World Health Organization [16], ranging from 10 to 19 years of age with or without sexual intercourse experience, and the parents/guardians who accompanied the adolescents.

A minimum representative sample size of 162 adolescents and 269 parents/guardians was calculated from the equation for a nonprobabilistic sample for an infinite population based on the proportion of 12% of 10–19 year olds and 50% of adult residents in the city of São Paulo in 2019 [17]. A confidence level of 95% (95% CI) and error size of 5% and 10% were used.

### Variables

The survey was composed of sociodemographic factors (sex, marital status, number of children, professional status, family monthly income, education) and had 24 questions measuring: 1- knowledge about HPV and its clinical repercussions; 2- knowledge about the HPV vaccine; 3- barriers to the HPV vaccination; and 4- HPV vaccine acceptability [12]. We chose to use the variable “sex” in our research according to the vaccination program of the Ministry of Health, which considers biological sex for vaccination. To participate in the study, adolescents were not required to answer this question; they had the option of not necessarily expressing sexual orientations and gender identities. We therefore consider that there was a greater inclusion of adolescents.

The answer options were “yes”, “no” and “I am not certain”. A score of (0) for incorrect answers and (1) for correct answers was attributed to each question. The proportions and confidence intervals for the correct answers were utilized to describe the correct answers for each question. For the score calculation, the numerator was the sum of correct answers multiplied by 100, and the denominator was the total number of answered questions. The questionnaire was tested on similar populations in a previous study [12]. The instrument had a Cronbach's alpha (α) value of 0.82, a very adequate score [18].

### Data sources

Adolescents and their parents/guardians were invited to voluntarily participate in the study and signed the written informed consent form and the consent form for adolescents under 16 years of age, when necessary.

The self-administered questionnaires were answered individually in the waiting room with no repercussions for health care. Previously trained postgraduate and undergraduate students were present in the same room to collect answers to the questionnaire. After completion of the instrument, counseling and education about HPV were provided.

### Statistical methods

The study population was divided into two groups: adolescents and their parents/guardians. The level of "suitable" knowledge about HPV, the vaccine and its clinical repercussions was the main endpoint of the study (i.e., the outcome).

Based on other studies [12,19] in which the authors used the same questionnaire and chose to analyze the answers using a cutoff point, we estimated the level of knowledge based on a pilot study with a sample of 20 parents/guardians and 20 adolescents. The total score of correct responses was calculated for each group. The parents answered correctly an average of 17 (70.6%) out of the 24 questions in the questionnaire. The adolescents, on the other hand, answered 11 of 24 (45.5%) questions correctly. Thus, a "suitable" level of knowledge was defined as a score ≥ 60% referring to the percentage of correct answers to items in the questionnaire “Knowledge and acceptance of HPV vaccine among adolescents, parents/guardians and health professionals” [12].

The following variables were considered explanatory variables: sex (male and female), age (10 to 14 years old, 15 to 19 years old, 20 to 39 years old, 40 to 59 years old, and over 60 years), marital status (single and stable relationship), childbirth (yes and no), education (less than high school, completed high school, undergraduate or higher), family monthly income (<USD 570.98, USD 570.98 to 1141.95, USD 1141.95 to 2854.88, and >USD 2854.88), and professional status (employed and unemployed). Stable relationship is defined as a relationship between two people who aim to start a family. We chose to use the variable “sex” in our study according to the vaccination program of the Ministry of Health, which considers biological sex for vaccination. An individual who does not fit this condition had the option of not responding”.

The explanatory or independent variables were expressed as the means of absolute frequencies and proportions. Less than 2% of the information was ignored or left blank and thus considered missing.

To analyze the association between the explanatory variables and "suitable" knowledge about HPV, clinical repercussions, and the vaccine and its acceptability, a Poisson regression model was performed by estimating the following measures: relative risk (RR) with a 95% confidence interval and the p value.

The univariate analysis was performed between a group of “adolescents and parents/guardians" and the correct answers to questions that were part of the questionnaire to estimate the following measures: relative risk (RR) with 95% confidence interval (95% CI) and the p value.

A multivariable adjusted model was constructed that included all variables that presented a p value <0.20 in the crude analysis. All analyses were performed using the STATA 15.1 program (College Station, TX, USA, 2018).

### Ethics statement

This study was approved by the University of Sao Paulo, Medicine School Research Ethics Committee (1.938.072). Adolescents and their parents/guardians were invited to voluntarily participate in the study and signed the Written Informed Consent Form and the consent form for adolescents under 16 years of age, when necessary.

## Results

A total of 1047 individuals participated in the study, of whom 74% (n = 776) were adolescents and 26% (n = 271) were parents/guardians. Both groups were composed of a majority of women, 69.5% (n = 530) among adolescents and 92.9% (n = 249) among parents/guardians. Among the adolescent participants, 64.9% (n = 504) were between 10 and 14 years of age, 98.6% (n = 714) were single, 4.5% (n = 33) had at least one child, 91.5% (n = 541) had less than a high school education and 36.2% (n = 258) had received at least one dose of HPV vaccine. Among the parents/guardians, 56.8% (n = 154) were between 40 and 59 years old, 65.4% (n = 149) were in a stable relationship, 73.1% (n = 190) had completed high school or higher, and 3% (n = 7) had received at least one dose of HPV vaccine (Table 1).

**Table 1:** Sociodemographic characteristics of adolescents and parents/guardians interviewed during the implementation of the HPV vaccine in 2015. Sao Paulo, Brazil, 2015.

Variables	Adolescents total n = 776	Parents/guardians total n = 271	Total n = 1047
	n (%)	n (%)	n (%)
**Sex**			
Female	530 (69.5)	249(92.9)	779 (75.6)
Male	233 (30.5)	19 (7.1)	252 (24.4)
**Age**			
From 10 to 14	504 (64.9)	-	504 (48.1)
From 15 to 19	272 (35.1)	-	272 (26.0)
From 20 to 39	-	99 (36.5)	99 (09.5)
From 40 to 59	-	154 (56.8)	154 (14.7)
Above 60	-	18 (6.7)	18 (1.7)
**Marital Status**			
Single	714 (98.6)	79 (34.6)	793 (83.3)
Stable relationship	10 (1.4)	149 (65.4)	159 (16.7)
**Childbirth**			
No	707 (95.5)	-	707 (69.9)
Yes	33 (4.5)	271 (100)	304 (30.1)
**Education**			
Less than High School	541 (91.5)	70 (26.9)	611 (71.8)
Complete HighSchool or higher	50 (8.5)	190 (73.1)	240 (28.2)
**Family Monthly Income**			
< USD 570.98	68 (19.7)	34 (17.1)	102 (18.7)
USD 570.98 to 1141.95	37 (10.7)	43 (21.6)	80 (14.7)
USD 1141.95 to 2854.88	30 (8.7)	83 (41.7)	113 (20.7)
> USD 2854.88	211 (60.9)	39 (19.6)	250 (45.9)
**Profession status**			
Employed	35 (6.7)	219 (91.6)	254 (33.5)
Unemployed	485 (93.3)	20 (8.4)	505 (66.5)
HPV Vaccination*			
Vaccinated	258 (36.2)	7 (3.0)	265 (27.9)
Non-vaccinated	455 (63.8)	228 (97.0)	683 (72.1)

*At least one dose.

**Fig 1:**
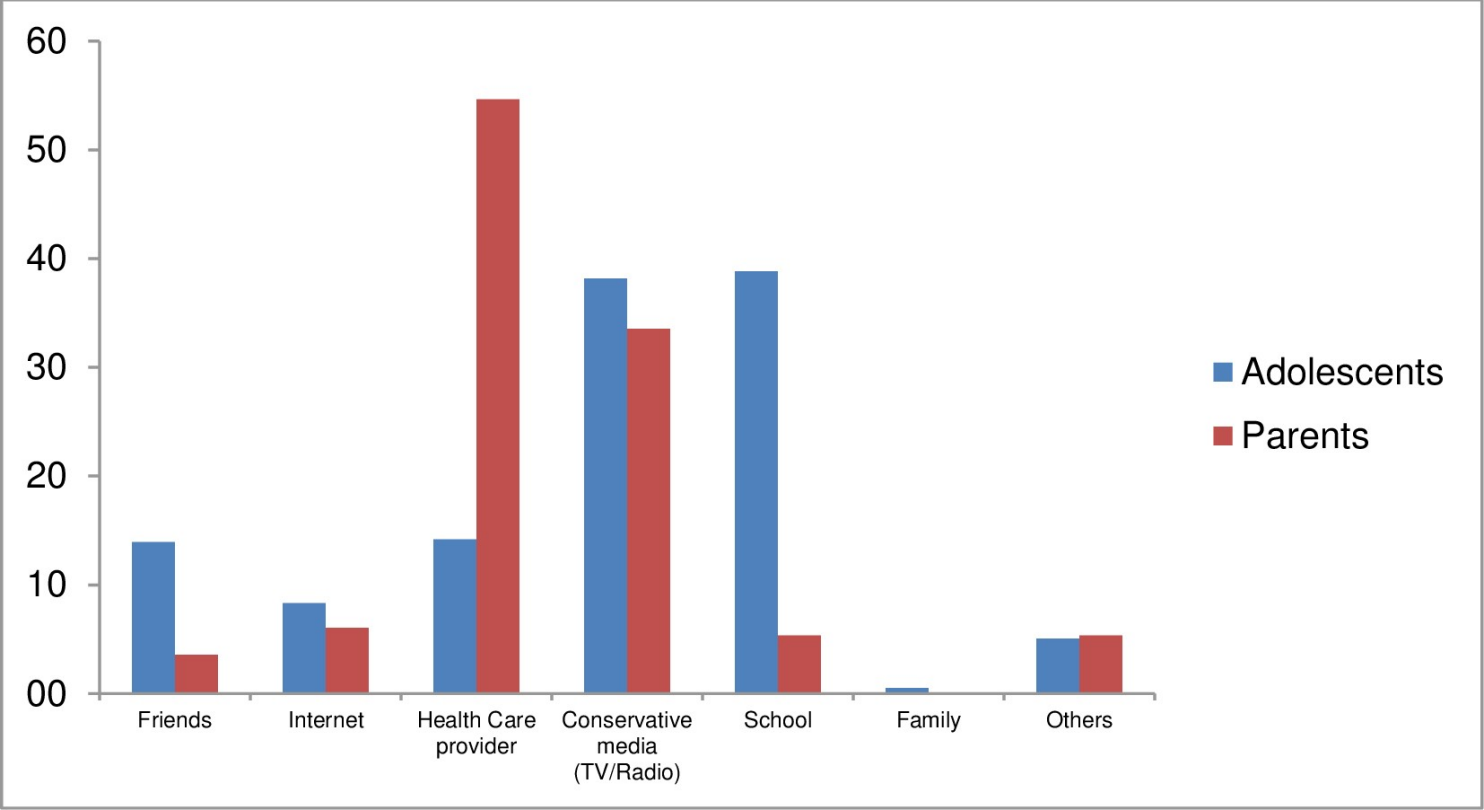
Main sources of information^1^ for the adolescents and parents/guardians interviewed during the implementation of the HPV vaccine in 2015. ^1^ As multiple answers were allowed, each was considered a dichotomous variable (yes/no).

The main source of HPV information among adolescents was school (39%, n = 298), followed by TV/radio (38%, n = 293). Parents/guardians acquired information about HPV mainly through health professionals (55%, n = 153), followed by TV/radio (33%, n = 94) (Fig 1).

Thus, questions answered correctly by less than 60% of the respondents were considered knowledge gap or barriers of acceptance, as shown in Tables 2 and 3. Still in Tables 2 and 3, the difference between adolescents and parents exists when one group reaches the threshold of 60% of correct responses and the other does not.

**Table 2:** Correct answers, differences in knowledge about HPV, its vaccine, acceptability and barriers to vaccination among adolescents and parents/guardians. Sao Paulo, Brazil, 2015.

Questions	Total Population Correct answers n (%)**	Adolescents Correct answers n (%)**		Parents/ Guardians Correct answers n (%)**		Crude RR (95% CI)		Interpretation		Differences among adolescents and parents	
Knowledge of HPV and its vaccine											
1. Do you know what HPV is?	610 (68.69)	372 (59.8)		238 (89.5)		1.39 (1.17–1.64)		Knowledge Gap of Adolescents		presence	
2. Is HPV a virus?	713 (69.09)	477 (62.0)		236 (89.7)		1.41 (1.20–1.65)				absent	
3. Is HPV a sexually transmitted disease?	713 (68.96)	468 (61.1)		245 (91.4)		1.43 (1.22–1.68)				absent	
4. Can HPV cause cervical cancer?	728 (70.13)	476 (61.8)		252 (94.0)		1.42 (1.21–1.66)				absent	
5. Can HPV cause changes in the Pap smear test?	438 (42.40)	230 (30.0)		208 (78.2)		2.48 (2.03–3.01)		Knowledge Gap of Adolescents		presence	
6. Is cervical cancer a major cancer in women?	707 (68.38)	468 (61.2)		239 (88.9)		1.38 (1.17–1.62)				absent	
7. Can smoking increase the risk of cervical cancer?	496 (47.74)	322 (41.8)		174 (64.7)		1.63 (1.34–1.98)		Knowledge Gap of Adolescents		presence	
8. Does the HPV vaccine prevent cervical cancer?	707 (68.38)	485 (63.2)		222 (83.2)		1.24 (1.05–1.46)				absent	
9. Should the HPV vaccine be given before the first sexual intercourse?	620 (59.79)	408 (53.1)		212 (78.8)		1.52 (1.27–1.81)		Knowledge Gap of Adolescents		presence	
10. Can the HPV vaccine be given to people who have had sex?	406 (39.23)	218 (28.3)		188 (70.9)		2.51 (2.05–3.08)		Knowledge Gap of Adolescents		presence	
11. Can the HPV vaccine be harmful to your health?*	543 (52.41)	328 (42.8)		215 (79.9)		1.79 (1.50–2.45)		Knowledge Gap of Adolescents		presence	
12. Can the HPV vaccine cause HPV infection?*	442 (43.38)	241 (32.1)		201 (75.3)		2.26 (1.86–2.75)		Knowledge Gap of Adolescents		presence	
13. Is the HPV vaccine provided by the government?	831 (79.90)	573 (74.3)		258 (95.9)		1.25 (1.07–1.46)				absent	
14. Is the HPV vaccine part of girls' immunization records?	618 (60.06)	461 (60.4)		157 (59.0)		0.93 (0.77–1.12)***		Knowledge Gap of Parents		absent	
15. Are 3 doses required for complete vaccination?	566 (57.17)	398 (53.6)		168 (67.7)		1.16 (0.97–1.40) ***		Knowledge Gap of Adolescents		presence	
16. Does the HPV vaccine decrease the chance of having genital warts?	350 (35.35)	179 (24.4)		171 (67.1)		2.77 (2.22–2.47)		Knowledge Gap of Adolescents		presence	
17. Does the HPV vaccine decrease the chance of having changes in the Pap smear test?	395 (39.66)	233 (31.4)		162 (64.0)		2.01 (1.63–2.48)		Knowledge Gap of Adolescents		presence	
HPV Vaccine’s Barriers											
18. Do you think the HPV vaccine will stimulate the onset of sexual activity at an earlier age?*	697 (70.55)		479 (64.9)		218 (87.2)		1.30 (1.11–1.55)		No barriers to vaccination		absent
19. Do you think that you still need to use a condom after HPV vaccination?	829 (82.98)		582 (78.2)		247 (96.9)		1.18 (1.01–1.38)		No barriers to vaccination		absent
20. Do you think that you still need to have a Pap smear test after HPV vaccination?	740 (74.75)		495 (67.3)		245 (96.5)		1.30 (1.11–1.52)		No barriers to vaccination		absent
Acceptability of HPV Vaccine											
21. Do you know anyone who has already received the HPV vaccine?	645 (66.84)		475 (65.3)		170 (71.4)		1.01 (0.84–1.21)***		Good acceptability		absent
22. Have you received the HPV vaccine yet?	265 (27.95)		258 (36.2)		7 (3.0)		0.65 (0.31–0.14)				absent
23. Would you recommend the HPV vaccine for a child, friend, or relative?	759 (79.64)		536 (74.9)		223 (93.7)		1.18 (1.01–1.39)		Good acceptability		absent

RR: Relative risk; 95% CI Confidence Interval; Poisson regression.

* Questions whose correct answer would be (No).

** The suitable level of knowledge considered is 60%.

*** p-value > 0.05.

**Table 3:** Multivariable analysis of factors associated with knowledge about HPV, its clinical repercussions and its vaccine among parents/guardians and adolescents interviewed during implementation of HPV vaccine. Sao Paulo, Brazil, 2015.

Variables	Adolescents	*P*-value	Parents/guardians	*P*-value
	RR (95% CI)		RR (95% CI)	
**Sex**				
Male	1.00		1.00	
Female	1.67 (1.10–2.60)	0.023	0.89 (0.46–1.70)	0.716
**Age**				
From 10 to 14	1.00		-	
From 15 to 19	1.39 (0.91–2.10)	0.126	-	
From 20 to 39	-		1.00	
From 40 to 59	-		1.11 (0.75–1.65)	0.603
Above 60	-		0.64 (0.23–1.80)	0.399
**Profission**				
Employed	1.00		1.00	
Unemployed	1.41 (0.69–2.88)	0.348	0.64 (0.22–1.83)	0.406
**Education**				
Less than High School	1.00		1.00	
High School Complete or higher	0.95 (0.48–1.91)	0.900	3.38 (1.71–6.69)	<0.001
**HPV Vaccination**				
Non-Vaccinated	1.00		1.00	
Vaccinated	1.89 (1.25–2.85)	0.002	1.33 (0.48–3.64)	0.578

RR: Relative risk; 95% CI Confidence Interval; Poisson regression.

Childbirth omitted because of collinearity.

The main knowledge gap in the parental group was that the question regarding whether the HPV vaccine would be part of girls' immunization records, with 59% (n = 157) correct answers. Among adolescents, only 24.4% (n = 179) had acceptable knowledge levels regarding the possibility that the HPV vaccine would decrease the chance of genital warts. Many adolescents expressed doubt that the HPV vaccine could be given to people who had previously had sexual intercourse, with a correct answer rate of 28.3% (n = 218) (Table 2).

Regarding the knowledge of HPV and its vaccine, the parents/guardians presented a higher level of knowledge for 15 of 17 questions on the subject. Parents/guardians were 2.48 times more likely than adolescents to know that HPV causes changes in the Pap smear test [RR 2.48, 95% CI 2.03–3.01 (p <0.001)], 1.43 times more likely to know that HPV was a sexually transmitted infection [RR 1.43, 95% CI 1.22–1.68 (p <0.001)] and 2.77 times informed that the HPV vaccine decreased the chance of having genital warts [RR 2.77, 95% CI 2.22–2.47 (p<0.001)] (Table 2).

No significant difference [RR 1.16; 95% CI 0.97–1.40 (p = 0.107)] was found in the answers to the question regarding the number of inoculations required for complete vaccination and the question regarding whether the HPV vaccine was part of girls' immunization records [RR 0.93; 95% CI 0.77–1.12 (p = 0.452)] (Table 2).

Both groups reached the satisfactory level of more than 60% correct answers regarding the barriers and acceptability (Questions 18–23) issues for the HPV vaccine. Adolescents were 0.65 times [RR 0.65; 95% CI 0.31–0.14 (p <0.001)] more likely to be vaccinated than their parents/guardians (Table 2).

Respondents with a stable marital status and a higher education level also presented significantly better results for knowledge and acceptability of HPV vaccine (RR 1.38; 95% CI 1.08 1.75 and RR 1.66; 95% CI 1.33–2.07, respectively) in the univariate analyses.

The multivariable analyses showed that girls in the adolescent group knew more about HPV than boys (RR 1.67; 95% CI 1.10–2.60) and vaccinated adolescents had more correct answers than nonvaccinated adolescents (RR 1.89; 95% CI 1.25–2.85). The category "high level of education" among parents/guardians corresponds to 3.38 times the number of correct answers to questions about HPV and its vaccine. [238% (RR 3.38; 95% CI 1.71–6.69)] (Table 3).

## Discussion

This study evaluated related factors and divergences in knowledge about HPV, its vaccine and acceptability among Brazilian adolescents and parents/guardians. The parents who accompanied their children to a reference unit for adolescent health knew more about these aspects than the adolescents. Information reached the young people mainly through the media and school, while the parents/guardians were informed through health professionals and the media. The factor associated with suitable knowledge in adolescents was the being female and in parents/guardians, was a high education level.

The adolescents presented low knowledge about HPV and its vaccine. The majority of our sample was composed of girls from 10 to 14 years old. Young people in this age group are the main target of the vaccination campaign for HPV in Brazil [6]. This population also deserves attention because these adolescents are experiencing a period of exposure to new experiences, physical changes and emotional instability, which makes them more vulnerable to contact with HPV and other sexually transmitted infections through unprotected sex [20,21]. Previous studies conducted in countries that adhered to vaccination showed that parents/guardians were the main source of information for their children [22,23]. Our results, on the other hand, showed that the family played a minimal role as a source of information for these adolescents. This reflects the reality of Brazilian families; even the ones with a good socioeconomic and education level, do not talk about this subject with their children. It is easier for Brazilian parents/guardians to transfer the responsibility of talking about sex education to school, mainly because they do not know how to approach the issue with their children [20,21].

The most common source of information among adolescents was school, followed by conservative media (TV/radio). This finding may be justified, because the first vaccination campaign for HPV in Brazil took place in public and private schools. Initially, lectures were given on the subject, and then vaccines were applied to the students after obtaining the parents/guardians' consent [6].

TV/radio media are an important source of information on the subject by reaching different age groups. This finding reinforces the promotion of qualified information bulletins to reduce myths and fake news about the HPV vaccine, which are mainly related to adverse events in the target population [24]. In addition, this approach has high potential to improve vaccination coverage in more remote areas of Brazil [25]. Moreover, the media managed to reach both adolescents and parents/guardians, as reported in previous studies [26].

Our findings showed that parents/guardians knew more about HPV and its vaccine and that a high level of education is a factor related to this knowledge. In addition, parents/guardians are more likely to know that HPV virus causes abnormal Pap smear results, since half of the parents/guardians are women, who are enrolled in cervical cancer screening (Pap smear) [27]. Similar results were found by Kose et al. (2014) [28], who addressed the knowledge levels of mothers of adolescents [28]. Other studies worldwide affirmed that a higher academic level was significantly associated with suitable knowledge about HPV [12,29].

The acquisition of knowledge depends on the experience ofthe subject as well as knowledge acquired during life. This occurs in the construction of both scientific knowledge and cultural knowledge [30]. Several studies conducted in countries with different cultures and vaccination policies corroborate our findings that adolescents had insufficient knowledge levels about HPV and its vaccine [31,32]. Furthermore, Brazilian adolescents do not receive sufficient sexual education at schools or home, which reinforces their lack of knowledge [33].

In this study, we identified that the adolescents did not know that the HPV vaccine could be given to people who had already had sex. The efficacy of the HPV vaccine is broadened when it is received before an individual first engages in sexual intercourse, but individuals who have had a sexual life can still receive the vaccine [34], knowing that the efficacy of the vaccine will be lower.

The only question the adolescents answered with greater accuracy than their parents/guardians was “Have you received the HPV vaccine yet?”. The reason for that might be the fact that adolescents over 16 years of age can be vaccinated without parental consent, especially those with HIV (free vaccination is available to individuals of either gender who are HIV positive, until 26 years of age) [6].

Young people older than the age covered by the Brazilian National Program of Immunization seek private services if they want to receive the vaccine. In addition, the National Health Surveillance Agency does not recommend applying vaccines to women out ofthe age range of 10 to 45 years and men from 9 to 26 years old [35].

This study points out that women tended to have an acceptable knowledge of HPV and its vaccine. Women were also consistently more knowledgeable than men in similar study [36]. The importance of reducing gender barriers is important mainly for men's health, as HPV also has clinical repercussions of genital warts and cancer ofthe penis, anus and oropharynx [5,37]. Given the divergences in knowledge between female and male adolescents, targeted strategies focusing on the male population should be prioritized for gender specific health educational actions.

The responses showed no barriers regarding HPV vaccination and good acceptance and willingness to recommend the vaccine. Although both the adolescents and their parents/guardians presented a suitable level of acceptability, the parents/guardians were significantly more likely to have no barriers than the adolescents. Because the parents/guardians had more knowledge and higher levels of education, we expected them to have lower rates of barriers [12,14]. A study carried out in Argentina prior to the implementation of HPV vaccine in the national immunization program also showed results favorable to its acceptability [38].

Although parents/guardians have a good level of knowledge, vaccination rates are still low in Brazil [7]. Reports of parents/guardians who do not vaccinate their children because of religious and cultural beliefs or myths surrounding vaccines are common, such as the rumor that the vaccine anticipates sexual activity in those who receive it [14].

HPV vaccination is a very effective public health policy for preventing cervical cancer. This policy is part of the National Immunization Program of the Brazilian Unified Health System [39,40]. However, during the HPV vaccination campaign implemented by the Brazilian Ministry of Health in 2014, negative media reports emerged about the adverse effects of the vaccine. This developed a feeling of insecurity in parents/guardians when deciding whether to vaccinate their children, resulting in reduced vaccination coverage. The results can help promote campaigns to enhance the understanding of parents/guardians and help them face their fears and beliefs.

The limitations of this cross-sectional study are that our sample was limited to adolescents and parents/guardians who were more likely to be interested in health issues, since they were recruited from a health center. Another limitation is that the outcome cannot be generalized to a different population, because it is a convenience sample. In addition, this limitation could be a source of bias in variable knowledge and perception, because this population analyzed individuals with access to health care, who did not represent the reality of other adolescents and parents/guardians in the Unified Health System. In this study, gender identity and sexual orientation were not considered, and we recognize that there is a gap in the research on these issues, which we will consider in future studies.

The highlights of this study include elucidating the divergences of knowledge among adolescents and parents/guardians and indicating parents/guardians' inability to pass on their knowledge and experiences experiences to their children. It can support sexual and reproductive health education programs to develop specific health promotion actions.

In fact, knowledge gaps and acceptance barriers are fundamental to the health education process and the social, cultural, and geographical contexts and aspects that integrate the individual and the community should be respected. According to the World Health Organization, prevention and primary health care depend on pillars in society, including the development of a country's education system [41]. Counseling and guidance disseminating current and true information facilitate the vaccine acceptance process, breach prejudices, and demystify inappropriate concepts about the HPV vaccine and can support aid with decision-making among adolescents and parents/guardians.

## Conclusion

Female gender, for either adolescents or parents/guardians was related to suitable knowledge about HPV and its vaccine among Brazilian respondents to the questionnaire. While adolescents and parents/guardians had differences in knowledge about HPV and its vaccine, there was no disagreement regarding vaccine acceptance. High-quality education materials adapted for both gender should be developed to provide comprehensive and detailed information about HPV and its potential health consequences. Furthermore, we highlight the need for health actions that facilitate the exchange of knowledge and experiences between parents/guardians and children, with an emphasis given to male adolescents with a low education level.
